# From sea to cure: Discovery of marine-derived therapeutics against *Fusarium solani* in shrimps for enhancing aquaculture sustainability

**DOI:** 10.1371/journal.pone.0336107

**Published:** 2025-10-31

**Authors:** Abdullah Al Siam, Avijit Kumer Paul, Shanjida Akter Joyoti, Md. Ifteker Hossain, Noimul Hasan Siddiquee, Bushra Binte Zaker, Al- Farabi, Shyamal Kumar Paul

**Affiliations:** 1 Department of Fisheries and Marine Science, Faculty of Biological Science, Noakhali Science and Technology University, Noakhali, Bangladesh; 2 Environmental Science Discipline, School of Life Science, Khulna University, Khulna, Bangladesh; 3 Department of Fisheries, University of Chittagong, Chittagong, Bangladesh; 4 Bioinformatics Laboratory (BioLab), Noakhali, Bangladesh; 5 Department of Microbiology, Faculty of Biological Science, Noakhali Science and Technology University, Noakhali, Bangladesh; 6 Department of Biotechnology, BRAC University, Dhaka, Bangladesh; Kwara State University, NIGERIA

## Abstract

*Fusarium solani*, an Ascomycota filamentous fungus species, causes shell disease or mycotic infections in wild and farmed shrimps. It causes black gill disease in shrimps, which has no specific treatments, so cutting-edge pharmaceutical research to prevent glutamine synthetase is needed to stop it and reduce its negative effects on aquaculture productivity and health. *In silico* drug design has been evaluated as an innovative treatment for black gill disease in shrimps caused by *F. solani*. Initially, molecular docking targeted the Glutamine synthetase (AF-Q9UUN6-F1-v4), utilising a set of 1,191 seaweed metabolites found in the Seaweed metabolite database (SWMD). The three lead compounds, CID: 359 (Phloroglucinol), 11640528 ((6E,10E,14E)-16-(2,5-dihydroxy-3-methylphenyl)-2-hydroxy-2,6,10,14-tetramethyl hexadeca-6,10,14-trien-3-one), and 8768 (Protocatechualdehyde), have binding affinities of −5.752, −5.374, and −5.102 kcal/mol, with negative binding free energies of −16.27, −48.99, and −27.48 kcal/mol, respectively. Additionally, they have excellent ADMET properties, making them safe and effective, whereas HOMO-LUMO and QSAR studies suggest thermodynamic stability and biological activity, notably antifungal efficacy. The compounds were subsequently assessed to verify their durability and binding affinity to the target protein by conducting an MD simulation analysis. In the MD simulation, the ligands evaluated in this study exhibited notable robustness of the proteins’ binding site when complexed with CID: 8768, which suggests a strong interaction between the target and lead compound. Consequently, the compound obtained from the seaweed *Polysiphonia lanosa* may inhibit the fungal activity of *F. solani* glutamine synthetase protein, revealing that the compound might be an effective novel therapeutic candidate.

## 1. Introduction

*Fusarium solani*, a diverse group of fungi with variable geographic distribution, pathogenicity, host/substrate, morphological traits, and homo- and heterothallic sexual phases, is widely used in plant pathology literature [1]. The FSSC has been extensively characterized in phylogenetic studies [2,3]. Currently, North American cultured lobsters and pond, tank, and raceway-reared penaeid prawns in Tahiti, Japan, and North and Central America are affected by *Fusarium* strains [4,5]. Despite only one major *Fusarium* disease outbreak in lobster cultivation, penaeid prawn farming in Japan and Mexico has suffered severe losses [6,7]. *F. solani* can lead to high rates of mortality in shrimp and is the causative agent responsible for shrimp black spot disease (BSD) [8,9]. Shrimp aquaculture is a rapidly growing, multi-billion-dollar industry essential for global food security and employment [10–12].

The *F. solani* species complex has genetic diversity, comprising many strains that demonstrate varying degrees of pathogenicity and virulence [13]. The genetic composition of *F. solani* is influenced by gene duplications, horizontal gene transfer, and genetic recombination, enhancing its adaptation to diverse environments [14,15]. The average genome size of *F. solani* isolates was 53.91 Mb, with the smallest measuring 45.81 Mb and the largest at 66.64 Mb. The GC content (%) varied between 49.5 and 51.5, whereas the mean protein length per genome ranged from 452 to 496, with a mean of 477 amino acids (AA). The FSSC includes opportunists, saprophytes, and pathogens that infect over a hundred different types of hosts, including humans, animals, and plants [16]. Because of their genetic diversity, this group of organisms can adapt to various settings, which makes them notable fungal pathogens [17].

The pathogenicity of *F. solani* involves extracellular enzymes, toxins, nitrogen assimilation, and biofilm development [14,18,19]. The capacity of *F. solani* to take advantage of the shrimp’s nitrogen absorption pathways is one of its primary virulence mechanisms. Fungal growth and the shrimp’s metabolism both depend on nitrogen, which the fungus may manipulate to its desired effect [20].

Studies have shown that targeting glutamine synthetase in fungal pathogens could reduce their ability to cause disease, as this enzyme is essential for nitrogen metabolism and detoxification of ammonia. In *F. solani*, this process supports fungal colonization and contributes to shrimp gill damage during black gill disease. Inhibiting this enzyme may potentially diminish the pathogenicity of *F. solani* [21]*.* This inhibition may interfere with the nitrogen metabolism of the host, which could make it easier for fungi to colonise and become pathogenic [22]. While infected, *F. solani* may create and excrete ammonia as a metabolic waste, mostly via urease enzymes that degrade urea in the shrimp’s tissues. Excess ammonia might overwhelm the shrimp’s ability to eliminate it [15]. High host tissue ammonia levels create a nitrogen-rich environment for *F. solani* to grow and survive. Glutamine helps the virus survive and replicate in prawn hosts by detoxifying ammonia. *F. solani’s* glutamine synthetase pathway assimilates ammonia, disrupting the prawn nitrogen balance [23,24]. However, prawns suffer from high ammonia levels, especially in the gills, where it disrupts cellular respiration. Black gill illness darkens the gills due to ammonia poisoning and fungal infection that damages the gill tissues [8,25].

The increasing worldwide shrimp production necessitates the urgent development of sustainable and effective treatments for black gill illness. For this investigation, we utilised the 1,191 seaweed metabolites, which were divided into 884 Red Algae (Rhodophyta), 33 Green Algae (Chlorophyta), and 274 Brown Algae (Phaeophyta) from the seaweed metabolite database rather than medicinal drugs. Seaweed is a significant source of bioactive compounds, including lipids, polyphenols, and polysaccharides. Due to their availability, productivity, and ease of extraction from nature, seaweed metabolites are a valuable resource for conducting rigorous clinical trials [26]. According to recent research, these chemicals exhibit a wide range of pharmacological properties, such as antioxidant, antibacterial, antiviral, anticoagulant, anticancer, and anti-inflammatory effects [27]. Seaweeds have enormous biotechnological promise for drug discovery due to their intrinsic flexibility, chemical richness, and capacity to alter their composition to generate targeted chemicals [28,29].

Computer-aided drug design (CADD) has become increasingly popular due to the increasing prevalence of *in-silico* chemistry and molecular modelling. This technique has proven to be both potent and effective in numerous contemporary therapeutic discoveries [30]. Our research employed docking and structure-based computational testing assays on the glutamine synthase protein to find potent inhibitors from a seaweed metabolite database. No control was chosen as there are no Food and Drug Administration (FDA)-approved drugs available. These metabolites of seaweed were evaluated against the glutamine synthetase protein of *F. solani* using bioinformatics tools, computer resources, and statistical analysis. Pharmacokinetic and toxicological property evaluations were used to further assess these antifungal medications’ potential. Additionally, in order to choose the most promising drug, molecular docking, ADMET, QM calculation, QSAR analysis, Post-docking MM-GBSA, and MD simulation were implemented to evaluate the durability and binding strength of the selected compounds in the glutamate synthase protease to ascertain the optimal pharmacological treatment.

## 2. Materials and methods

### 2.1 Preparation of the desired protein and ligand

Using the AlphaFold Protein Structure Database (https://alphafold.ebi.ac.uk/), the 3D configuration of the Glutamine synthetase (AF-Q9UUN6-F1-v4) of the *F. solani* was isolated. It has a length of 358 AA and is composed of a single chain (Chain A), with a pLDDT score of > 90. To maximise further research, the protein structure was prepared using Maestro 13.5’s protein preparation program. Therefore, the protein’s bond ordering and the addition of hydrogen atoms and side chain residues were accomplished using the default setting for protein macromolecule processing. Protein preparation was carried out using the OPLS4 force field [31,32].

This study investigated 1,191 seaweed metabolites, which were divided into 884 Red Algae (Rhodophyta), 33 Green Algae (Chlorophyta), and 274 Brown Algae (Phaeophyta). 1,191 seaweed metabolites with antifungal properties were analysed to find promising antagonists. The natural compounds were extracted from the Seaweed Metabolite Database (SWMD) (https://www.swmd.co.in/) in SDF file format. Consequently, the LigPrep module of Maestro 13.5 was used for developing ligands, and protein-molecule structures were optimised with the aid of the OPLS4 force field [31].

### 2.2 The inspection of molecular docking

Molecular docking is an excellent screening tool that aims at determining the best binding capability and linking configuration for the ligand and the target [33]. This method can provide important insights into the relationships between ligand-protein (L-P) and other physiological processes, as well as the development of new medications [34]. The procedure includes docking 1,191 seaweed metabolites with the target protein using Schrödinger-Desmond tools (Maestro 13.5 and Glide v8.8 modules). The OPLS4 force field in standard precision mode was employed for the whole docking procedure [31]. The complete surface of the target protein was included in the receptor grid generation in order to study the native inhibitor in conjunction with the target protein and perform blind docking. A box with the coordinates (X = 0.832 **Å**, Y = 1.97 **Å**, and Z = 0.026 **Å**) was employed, along with information on the residues in the receptor’s binding sites. The energy of ligands binding to the target protein was measured, and the Maestro viewer exhibited various chemical bond types and residues that interact with ligands.

### 2.3 Inspection of pharmacokinetics (PK) and toxicity (ADMET) analysis

Pharmacokinetics is formed from two terms: pharmaco (meaning “drug”) and kinesis (meaning “movement”). By examining how foreign substances, like drug-like compounds, move dynamically throughout the body, their fate can be deciphered, comprehended, and even anticipated [35]. Therefore, mathematical formulas are used in quantitative research involving medication ADME rates to comprehend fundamental concepts and even assess the variety and spectrum of toxic or medicinal outcomes [36]. Additionally, this has a significant and early impact on drug design, influencing the medication’s efficacy and safety. This work evaluated the PK characteristics of targeted compounds by applying the Swiss Institute server SwissADME (http://www.swissadme.ch/) to evaluate the behaviour of every constituent of the seaweed metabolites [37].

The enhancement of drug-like characteristics of top compounds, together with the improvement of their pharmacokinetics, safety, and efficacy through the evaluation of toxicity throughout drug development, is essential for progress, success, and therapeutic effectiveness [38]. This involves employing sophisticated computational tools and predictive models via the ProTox-3.0 platform (https://tox.charite.de/protox3/) for the forecasting of chemical toxicity, utilising a validated web tool with an established and generally adopted approach [39]. However, we have conducted an ADMET (Pharmacokinetics and toxicity) study on the top five compounds with higher docking results.

### 2.4 Post-docking MM-GBSA evaluation

MM-GBSA is an enhanced virtual method commonly utilised to calculate the free energy of protein-ligand interactions. This approach is especially valuable for binding studies of biological macromolecules and ligands [40,41]. Using Maestro 13.5 and the Prime MM-GBSA v3.0 module, we visually examined and analysed the lowest energy binding conformations of compounds. Docking and MM-GBSA scores were used as control factors for comparison with newly tested drugs. A decreased MM-GBSA point describes a much more stable binding structure [42]. The docked pose viewer file was used to calculate the MM-GBSA values of the ADMET-passed top compounds, which provides a more appropriate scoring function for complex systems.

### 2.5 Quantum mechanical (QM) calculation

Quantum mechanical (QM) Time-dependent density functional theory (TD-DFT) techniques were used to calculate the ligands’ binding locations and their interactions with proteins. TD-DFT was used to optimise each compound using Lee, Yang, and Parr’s (LYP) correlation functional and Becke’s (B) three-parameter hybrid model under Pople’s 6-31G basis set [43–46]. In this study, calculating frontier molecules’ orbital properties, such as ∊HOMO (Highest Occupied Molecular Orbital) and ∊LUMO (Lowest Unoccupied Molecular Orbital), was done utilising frontier molecular orbital theory for the lead compounds. The energies of the frontier HOMOs and LUMOs were also utilized to calculate the HOMO-LUMO gap (ΔE), hardness (η), softness (S), and chemical potential (μ) for each of the compounds using the Parr and Pearson interpretation of DFT and Koopmans’ theorem [47,48].


$$Gap\;(\Delta E)=[\varepsilon LUMO-\varepsilon HOMO];\;\eta \;=\;\frac{\left[\epsilon LUMO-\epsilon HOMO\right]}{\text{2}};\;S\;=\frac{\text{1}}{2\eta };\mathrm{\backslash ]}$$

$$\mu \;=\frac{\left[\epsilon LUMO+\epsilon HOMO\right]}{\text{2}}\mathrm{\backslash ]}$$

### 2.6 Quantitative structure-activity relationship (QSAR) analysis

The biological activity spectrum of the thymidine esters was predicted using the Prediction of Activity Spectra for Substances (PASS) online program (http://www.pharmaexpert.ru/PASSonline/index.php). In this research, the PASS online program was used to forecast the biological processes of the lead compounds’ structures. With an accuracy of over 90%, PASS can identify the most likely biological targets and predict over 4,000 different biological activities, including both drug and non-drug actions [49]. With values ranging from 0.000 to 1.000, the prediction outcomes are represented as Pa (probability of activity) and Pi (probability of inactivity). Given that both probabilities are computed independently, it is crucial to remember that Pa + Pi ≠ 1. The chemical is thought to have potential for just those actions for which Pa > Pi. The PASS results are interpreted as follows: (i) if Pa > 0.7, there is a high chance that the activity will be verified experimentally; (ii) if 0.5 < Pa < 0.7, there is a low chance of experimentally verifying the activity, but the compound may not resemble known pharmaceuticals; and (iii) if Pa < 0.5, there may be a structural similarity to existing pharmaceutical agents, but the probability of experimentally verifying the activity is even lower [50]. Therefore, a compound’s inherent features are reflected in the prediction of its biological activity spectrum.

### 2.7 MD simulation analysis

MD simulation is a virtual technique that examines the physical and chemical features of molecules and atoms employing a classical force field, which integrates parameter sets and functional forms for the determination of the potential energy of the system’s components [51]. It elucidates the time-dependent progression of a completely dissolved molecular system with atomic precision, achieved by the numerical determination of Newton’s second law of motion [52]. The protein-ligand (P-L) complexes were initially constructed through molecular docking, which was subsequently followed by pre-processing using the Protein Preparation Wizard [53]. Therefore, the durability of the P-L complex was assessed by 100 ns MD simulations that tested the binding affinity of the chosen chemical to the target protein, applying the Desmond package from the Schrödinger suite. In order to ensure that the system volumes remain constant, an orthorhombic periodic boundary box with a dimension of (10 × 10 × 10 **Å**^3^) has been set up for each complex SPC water model [42]. While maintaining a salt concentration of 0.15 M, the solvated system was arbitrarily inundated with Na^+^ and Cl^-^ ions. A force field, the OPLS4, was implemented to ease and unwind the system [54]. Subsequently, an equilibration was conducted on each complete system to guarantee the system’s resilience through NPT ensemble performance at a controlled pressure and temperature of 1.01325 bar and 300K, followed by energy 1.2 recorded intervals of 100 ps. The solvent and ions had been distributed uniformly throughout the protein-ligand complex at this stage [55].

## 3. Result

### 3.1 The evaluation of molecular docking

**Fig 1:**
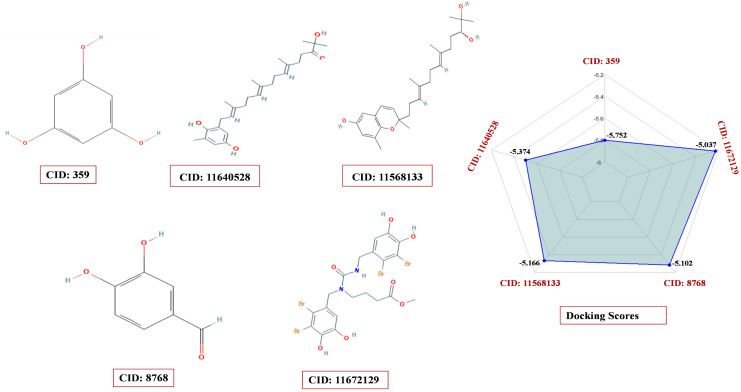
3D representations of the top five seaweed-derived compounds docked to *Fusarium solani* glutamine synthetase (AF-Q9UUN6-F1-v4). (A) Phloroglucinol (CID 359); (B) CID 11640528; (C) CID 11568133; (D) Protocatechualdehyde (CID 8768); (E) CID 11672129.

A molecular docking study was performed to elucidate the molecular relationships and binding abilities between the seaweed metabolites and the target protein, using 1,191 compounds for assessment employing the Schrödinger suite’s Maestro module. Among these, we have chosen five compounds, CID: 359 (BE001), CID: 11640528 (BS015), CID: 11568133 (BS041), CID: 8768 (RP004), and CID: 11672129 (RR017) (Table 1), for further analysis (ADME and toxicity) as they have a higher binding affinity with docking values −5.752, −5.374, −5.166, −5.102, and −5.037 kcal/mol, respectively (Fig 1).

**Table 1:** List of the top five compounds (CIDs: 359, 11640528, 11568133, 8768, and 11672129), with their PubChem ID, names, sources, and therapeutic uses.

PubChem CID	Compound Name	Source (Seaweed metabolites)	Therapeutic Use
**CID: 359**	Phloroglucinol	*Ecklonia stolonifera* [34]	Anti‑inflammatory, Anticancer [56] Antibacterial, Antifungal, Antiviral [57] Antioxidant [58,59]
**CID: 11640528**	(6E,10E,14E)-16-(2,5-dihydroxy-3-methylphenyl)-2-hydroxy-2,6,10,14-tetramethylhexadeca-6,10,14-trien-3-one	*Sargassum micracanthum* [59]	Antioxidant and cytotoxic against cancer [59]
**CID: 11568133**	(3R,6E,10E)-13-(6-hydroxy-2,8-dimethyl-chromen-2-yl)-2,6,10-trimethyl-trideca-6,10-diene-2,3-diol	*Sargassum fallax* [60]	Antitumour [61,62] Anticancer [63]
**CID: 8768**	Protocatechualdehyde	*Polysiphonia lanosa* [64]	Antimicrobial, Antioxidant, and Anti-inflammatory [65]
**CID: 11672129**	Methyl N,N’-bis-(2,3-dibromo-4,5-dihydroxybenzyl)-Y-ureidobutyrate	*Rhodomela confervoides* [60]	Antioxidant [61] Anti-cancer [66]

### 3.2 Review of pharmacokinetics (PK) and toxicity traits (ADMET)

Through the use of ADME attributes, which include procedures that are essential to comprehending the PK of a drug as a whole, the pharmacokinetic profile of the medicine can be improved. On the basis of the high docking score, we evaluated the ADME properties of five natural compounds that were among the 1,191 docked molecules inside the SWMD database. Accordingly, the study used the SwissADME service to examine the PK properties of the five chosen drug-like compounds (CID: 359, 11640528, 11568133, 8768, and 11672129).

Toxicology evaluates a substance’s potential for harm to organs and its degree of toxicity to humans [67]. One crucial stage of *in-silico* drug development is determining a molecule’s toxicity, which can be achieved by using the ProTox-3.0 server. The characteristics of the compounds are categorised into toxicity endpoints and organ toxicity.

**Fig 2:**
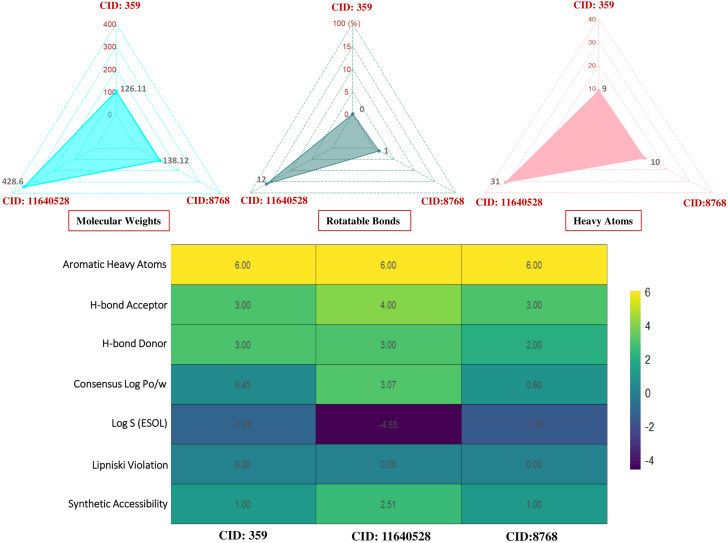
Pharmacokinetic (PK) properties of the three lead seaweed metabolites (CID 359, 11640528, and 8768) predicted using the SwissADME server. Parameters shown include lipophilicity (LogP), molecular weight, hydrogen bond donors/acceptors, and topological polar surface area, evaluated under Lipinski’s rule of five criteria.

**Fig 3:**
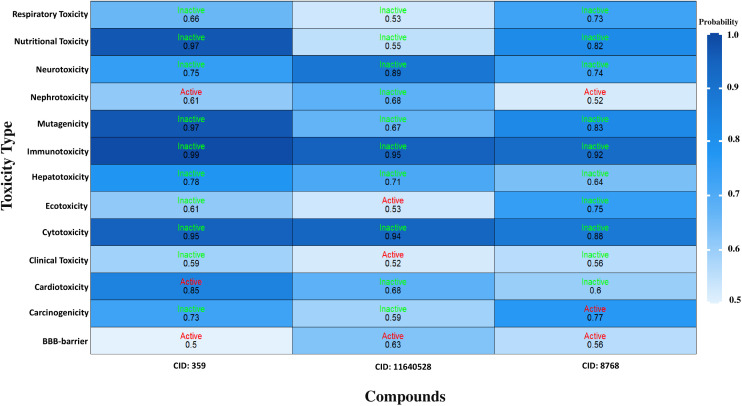
Toxicity profiling of the three lead compounds (CID 359, 11640528, and 8768) assessed. The heatmap depicts toxicity endpoints, including hepatotoxicity, mutagenicity, carcinogenicity, and immunotoxicity, along with organ-specific toxicity predictions.

Consequently, three compounds (CID: 359, 11640528, and 8768) were chosen for further studies after the ADMET analysis because they met all five of Lipinski’s criteria and had favourable toxicity characteristics in addition to potential drug-like qualities (Figs 2 and 3).

### 3.3 Protein-ligand interaction analysis

**Fig 4:**
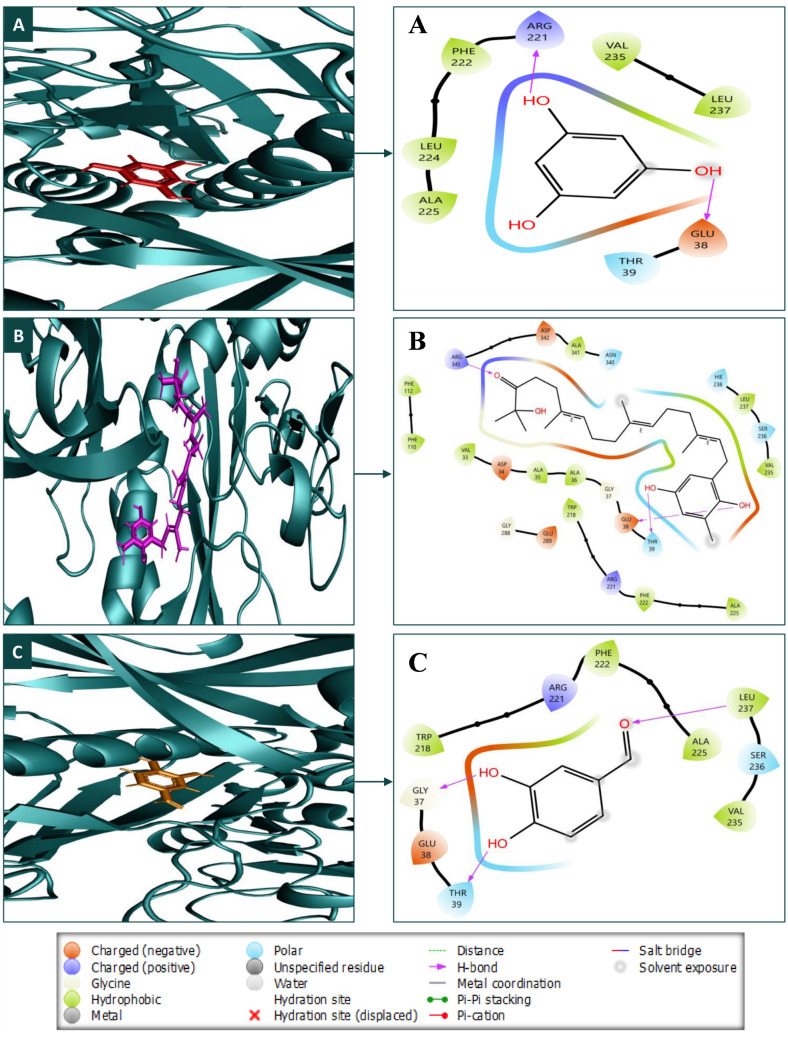
The relationship between the *F. solani* glutamine synthetase protein and three lead compounds in 3D and 2D formats, with compounds (A) CID: 359, (B) CID: 11640528, and (C) CID: 8768 in the protein’s active site.

For further analysis, the three compounds with the most favourable ADMET characteristics and no notable violations were chosen to display their molecular relationships with the target protein employing the Maestro module of the Schrödinger suite. During molecular docking (Table 2), the three lead compounds engaged with the protein’s common AA residues, and the interactions of the compounds, identified by their PubChem CIDs (359, 11640528, and 8768), with various amino acid residues through hydrogen, polar, and hydrophobic bonds. CID: 359 forms hydrogen bonds with ARG221 and GLU38, polar interactions with THR39, and extensive hydrophobic interactions, including residues like LEU237 and PHE222. CID: 11640528 exhibits a broader range of interactions, forming hydrogen bonds with GLU38, THR39, and ARG345, and polar interactions with multiple residues, such as THR39, ASN340, and SER236, while engaging hydrophobic residues like VAL235. CID: 8768 shows hydrogen bonding with residues like LEU237 and THR39, polar bonds with THR39 and SER236, and hydrophobic interactions involving PHE222 and ARG221(charged residue). The amino acids that are commonly found are GLU38 and THR39 (polar bond). THR39 is commonly found in polar bonds. As detailed in Table 2 and depicted and discussed in Fig 4, each of the three compounds that were chosen operated in tandem with a distinct Amino Acid (AA). Fig 4 displays the molecular interactions of a ligand with its surrounding residues in three different binding modes (A, B, and C). Each panel highlights key interactions such as hydrogen bonds (purple dashed lines), hydrophobic contacts, salt bridges, and polar associations. The residues are categorised based on their properties (e.g., hydrophobic, polar, charged) using colour coding. The ligand’s coordination with the protein is shown with annotations, focusing on specific residues like ARG 221, PHE 222, GLU 38, and LEU 237.

**Table 2:** Emphasising the amino acid residues linked to hydrophobic, polar, and hydrogen bonds that are created between the target protein and the three chosen drug-like compounds.

PubChem CID	Hydrogen Bonds	Polar Bonds	Hydrophobic Bonds
**CID: 359**	ARG221, GLU38	THR39	LEU237, VAL235, PHE222, LEU224, ALA225, GLU38,
**CID: 11640528**	GLU38, THR39, ARG345	SER236, HIE238, ASN340, THR39	VAL235, SER236, LEU237, HIE238, ALA341, ASP342, ARG345, PHE110, VAL33, ASP34, ALA35, ALA36, GLY37, GLU38
**CID: 8768**	LEU237, THR39, GLY37	THR39, SER236	PHE222, TRP218, GLY37, GLU38,

### 3.4 Post-docking MM-GBSA study

**Fig 5:**
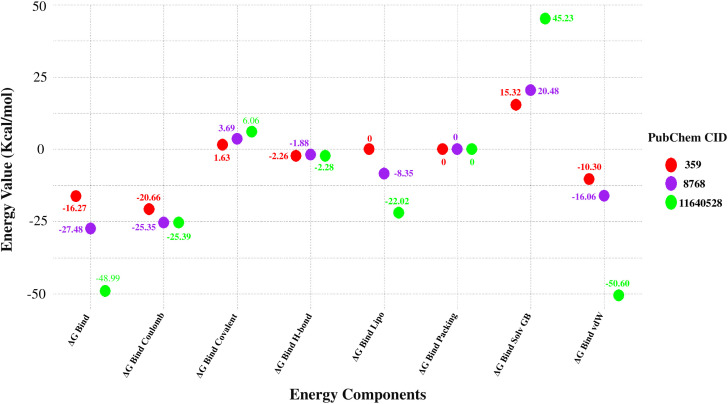
Binding free energy (ΔG_bind) profile of the three lead compounds derived from post-docking MM-GBSA analysis. Each dot represents the total binding free energy (kcal/mol), with lower values indicating higher binding stability to the glutamine synthetase active site.

The MM-GBSA technique was employed to determine the free energy of binding at the P-L complex endpoint [41]. The selected compounds (CID: 359, 11640528, and 8768) interacted with the target protein, exhibiting negative ΔG_bind values consistent with stable binding and an overall negative trend, as determined by the MM-GBSA study. After molecular docking, molecules CID: 359, 11640528, and 8768 showed negative binding free energies of −16.27, −48.99, and −27.48 kcal/mol, respectively. Out of all these compounds, CID: 11640528 had the most negative binding free energy. Based on ΔG_bind classification, CID: 11640528 was identified as a strong binder, CID: 8768 as a moderate binder, and CID: 359 as a weak binder. Subsequent examination of these three lead compounds revealed that ΔG Bind Coulomb, ΔG Bind Lipo, ΔG Bind Solv GB, ΔG Bind Hbond, ΔG Bind Packing, and ΔG Bind vdW contributed differentially to binding, where Coulombic, van der Waals, and lipophilic interactions favored stability, while solvation energy showed unfavorable contributions. The extensive evaluation of MM−GBSA also determined −20.66, −25.39, and −25.35 kcal/mol of ΔG Bind Coulomb; 0, −22.02, and −8.35 kcal/mol of ΔG Bind Lipo; 15.32, 45.23, and 20.48 kcal/mol of ΔG Bind Solv GB; −2.26, −2.28, and −1.88 kcal/mol of ΔG Bind H-bond; 0, 0, and 0 kcal/mol of ΔG Bind Packing; −10.30, −50.60, and −16.06 kcal/mol of ΔG Bind vdW for the molecules CID: 359, 11640528, and 8768, respectively. (Fig 5). The data shown above suggests that the three selected compounds may impede the target macromolecule. These results confirm that CID: 11640528 exhibits the strongest binding potential, while CID: 8768 and CID: 359 demonstrate moderate and weak affinities, respectively.

### 3.5 Quantum mechanical (QM) calculation

**Fig 6:**
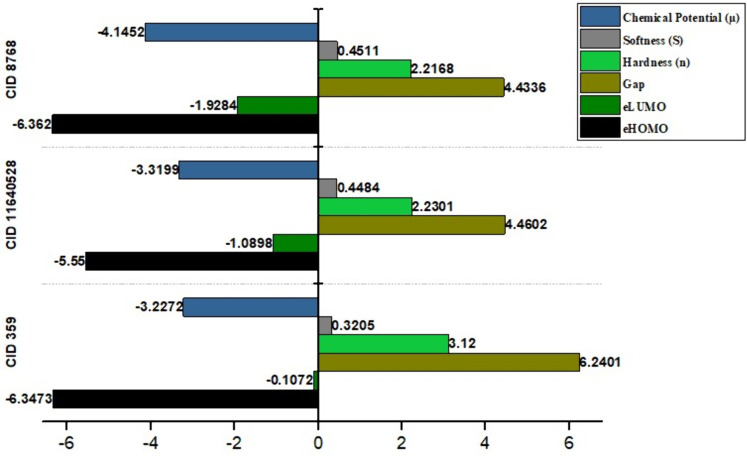
HOMO-LUMO value, Chemical potential, Softness, and Hardness of three chosen compounds CID: 359, 11640528, and 8768.

**Fig 7:**
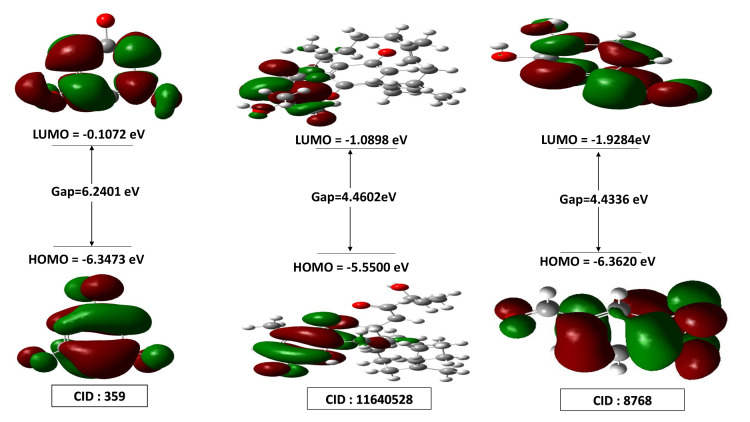
HOMO-LUMO energy gap of three chosen compounds CID: 359, 11640528, and 8768.

By electron excitation from the HOMO to the LUMO, the frontier molecular orbital theory offers information on a molecule’s chemical reaction potential, specifically corresponding to the HOMO and LUMO [43,44,68]. The electrophilic index, chemical potential values, softness, and hardness of a molecule are all influenced by the HOMO-LUMO energy [44]. A low HOMO-LUMO gap is associated with lower chemical hardness, higher chemical softness, less kinetic stability, and better chemical reactivity and polarisability. However, they are less likely to be photochemically active due to their high HOMO-LUMO gap, which causes significant kinetic durability and low chemical responses. Consequently, CID: 359 has the largest energy gap (6.2401 eV), the greatest chemical hardness (3.12 eV), and the lowest chemical softness (0.3205 eV^-1^) than the other two compounds (CID: 11640528 and 8768). Both compounds, CID: 11640528 and CID: 8768, have the lower energy gap (4.4602 eV and 4.4336 eV) and chemical hardness (2.2301 eV and 2.2168 eV), but the highest chemical softness (0.4484 and 0.4511 eV^-1^). The lead compounds demonstrate chemical reactivity and stability in the following order: CID: 359 > CID: 11640528 > CID: 8768. Although all of them are prospective candidates for drug design, CID: 11640528 and 8768 exhibit greater reactivity as a result of their HOMO-LUMO gap values, rendering them more suitable as potential drug candidates, as depicted in Figs 6 and 7. These observations are relevant because a smaller HOMO–LUMO gap is associated with higher chemical reactivity and a stronger likelihood of interaction with biological targets such as fungal enzymes. The lower energy gap values of CID: 11640528 and 8768, therefore, support their potential antifungal activity, as they may form more favorable electronic interactions with *F. solani* glutamine synthetase compared to CID: 359.

### 3.6 Quantitative structure-activity relationship (QSAR) analysis

The bioactive chemicals in the extract with a wealth of medicinal potential will be identified with the use of the PASS study. With the aim of finding new drugs, PASS has been used as a potent perspective tool to predict the biological function spectrum of synthetic compounds [69]. Table 3 presents the results of the QSAR analysis, evaluating the biological function spectrum of three seaweed-derived compounds for potential medicinal applications. Each compound is assessed for specific predicted activities using the PASS tool, with probabilities of activity (Pa) and inactivity (Pi) reported. Phloroglucinol (CID: 359) exhibits high probabilities of activity in several roles, including as an Aspulvinone dimethylallyltransferase inhibitor (Pa = 0.954) and a testosterone 17beta-dehydrogenase inhibitor (Pa = 0.919). These activities suggest potential applications in modulating membrane integrity and metabolic pathways. Its antiseborrheic and antifungal potentials (Pa > 0.931) are particularly notable, emphasising its dermatological and therapeutic utility. The compound (CID: 11640528) shows strong reductant activity (Pa = 0.948) and moderate antifungal potential (Pa = 0.575). Its role as a lipid peroxidase inhibitor (Pa = 0.825) highlights potential antioxidant benefits, while its inhibition of CDP-glycerol glycerophosphotransferase (Pa = 0.802) and antipsoriatic effects (Pa = 0.841) suggest applications in treating skin conditions. Protocatechualdehyde (CID: 8768) demonstrates notable activities as an aldehyde oxidase inhibitor (Pa = 0.954) and a feruloyl esterase inhibitor (Pa = 0.923), underscoring its role in metabolic and oxidative processes. Its ability to inhibit benzoate 4-monooxygenase and L-glucuronate reductase (Pa > 0.880) may have implications in metabolic engineering and antifungal therapies.

**Table 3:** The outcomes of the QSAR models that estimate the bioactivity of the selected compounds.

PubChem CID	Compound Name	Pa	Pi	Activity
**CID: 359**	Phloroglucinol	0.954	0.003	Aspulvinone dimethylallyltransferase inhibitor
		0.941	0.004	Membrane integrity agonist
		0.931	0.003	Antiseborrheic
		0.932	0.006	CYP2C12 substrate
		0.919	0.004	Testosterone 17beta-dehydrogenase (NADP+) inhibitor
**CID: 11640528**	(6E,10E,14E)-16-(2,5-dihydroxy-3-methylphenyl)-2-hydroxy-2,6,10,14-tetramethylhexadeca-6,10,14-trien-3-one	0.948	0.002	Reductant
		0.825	0.003	Lipid peroxidase inhibitor
		0.841	0.021	CDP-glycerol glycerophosphotransferase inhibitor
		0.802	0.004	Antipsoriatic
		0.575	0.021	Antifungal
**CID: 8768**	Protocatechualdehyde	0.954	0.003	Aldehyde oxidase inhibitor
		0.923	0.003	Feruloyl esterase inhibitor
		0.914	0.002	Benzoate 4-monooxygenase inhibitor
		0.880	0,003	L-glucuronate reductase inhibitor
		0.879	0.014	CDP-glycerol glycerophosphotransferase inhibitor

In particular, the predicted antifungal-related activities (Pa > 0.5) are relevant because they indicate a statistically meaningful likelihood of biological effect under the PASS model. Such probabilities strengthen the case that these compounds could interact with fungal targets in a biologically relevant manner, supporting the antifungal potential inferred from docking and QM results. These predictions underline the compounds’ promise as antifungal agents, among other uses. Their high Pa values suggest reliability in their predicted activities, warranting further experimental studies and MD simulations to validate and explore their medicinal applications, particularly in drug discovery efforts targeting fungal infections.

### 3.7 MD simulation analysis

**Fig 8:**
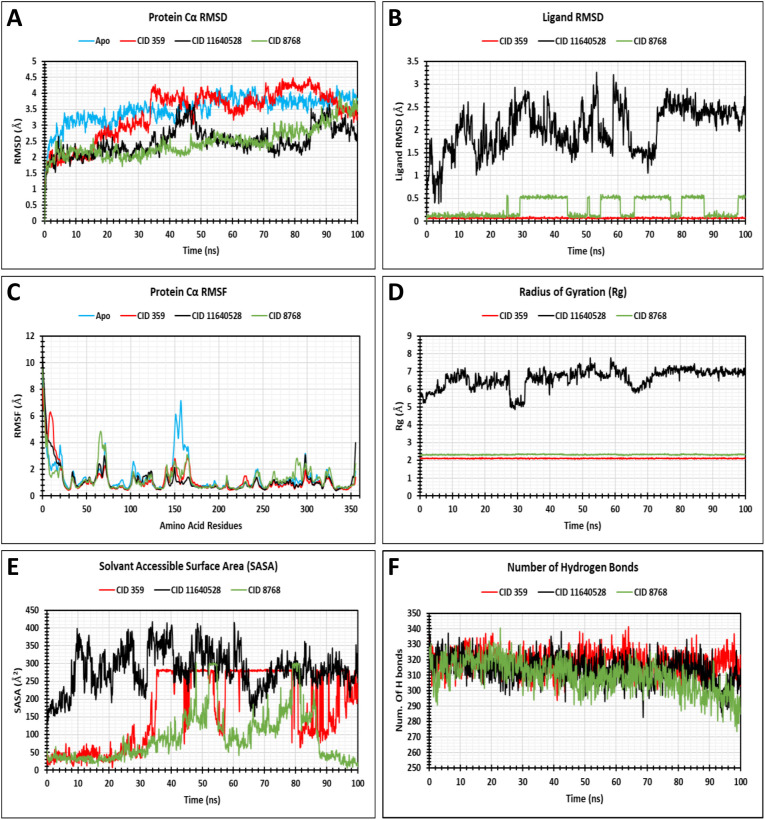
The findings for protein RMSD (A), ligand RMSD (B), RMSF (C), Rg (D), SASA (E), and H-bond (F) retrieved from data analysis trajectories employing the MD simulation techniques.

In order to gain more insight into the structural alterations of the protein in response to a specific ligand, a 100 ns MD simulation was carried out. The MD simulation produced 1000 frames at 100 ps intervals for trajectory recording and examined the trajectory file to assess protein and ligand root mean square deviation (RMSD), protein root mean square fluctuation (RMSF), radius of gyration (Rg), solvent-accessible surface area (SASA), and protein-ligand (P-L) contacts analysis using the simulation interaction diagram (SID), and the simulation event analysis was employed to conduct the H-bonds analysis. (Fig 8).

#### 3.7.1 Protein root mean square deviation (RMSD) analysis.

In molecular modelling, the RMSD (Root Mean Square Deviation) is a metric that is used to evaluate the architectural robustness between proteins [70]. It is essential for determining the structural durability of proteins or protein-ligand complexes over a certain time period [71]. The protein’s equilibrium state can be measured by examining the decline of the RMSD graph and the simulation’s consistent fluctuation, and the relatively narrow range of RMSD scores indicates that the protein foundation is showing firmness [42]. Therefore, a 100 ns MD simulation was performed to explore the conformational alteration of the target macromolecule in the complex with the chosen compounds, comprising CID: 359, 11640528, and 8768, as shown in Fig 8A. The average RMSD of target protein was 3.504 **Å**, indicating instability during the simulation period, and the most notable fluctuations emerged throughout the simulation’s 5–20 ns, 36 ns, and 74–88 ns. For the target protein, the highest and lowest RMSD scores were 4.247 **Å** and 1.221 **Å**, observed at frame numbers 596 and 1, respectively. However, CID: 11640528 and 8768 exhibited outstanding stability when complexed with the target protein, with slight fluctuations observed during the simulation time, in contrast to CID: 359. The mean RMSD values for CID: 359, 11640528, and 8768 were 3.364 **Å**, 2.484 **Å**, and 2.466 **Å**, respectively. The highest RMSD values of the three compounds, CID: 359, 11640528, and 8768, were 4.508 **Å**, 3.651 **Å**, and 4.048 **Å**, observed at frame numbers 847, 462, and 943, correspondingly, while the smallest were 1.16 **Å**, 0.955 **Å**, and 0.98 **Å**. observed at frame numbers 1, 3, and 1 (Table 4).

**Table 4:** The highest, lowest, and average values of Protein RMSD, Ligand RMSD, Protein RMSF, Rg, SASA, and H-bond.

Parameter	Value	CID: 359	CID: 11640528	CID: 8768
**Protein Cα RMSD**	Highest value (**Å**)	4.508	3.651	4.048
	Lowest value (**Å**)	1.16	0.955	0.98
	Average value (**Å**)	3.36	2.48	2.47
**Ligand RMSD**	Highest value (**Å**)	0.111	3.259	0.586
	Lowest value (**Å**)	0.039	0.385	0.059
	Average value (**Å**)	0.07	2.03	0.29
**Protein Cα RMSF**	Highest value (**Å**)	8.089	10.883	9.53
	Lowest value (**Å**)	0.442	0.39	0.485
	Average value (**Å**)	1.183	1.184	1.29
**Radius of gyration**	Highest value (**Å**)	2.148	7.782	2.382
	Lowest value (**Å**)	2.064	4.857	2.252
	Average value (**Å**)	2.11	6.62	2.32
**Solvent-accessible surface area**	Highest value (**Å**^**2**^)	283.964	417.765	302.789
	Lowest value (**Å**^**2**^)	6.621	134.33	4.678
	Average value (**Å**^**2**^)	170.84	283.22	91.85
**No. of Hydrogen Bonds**	Highest value	341	338	340
	Lowest value	294	283	274
	Average value	318.21	313.68	309.35

#### 3.7.2 Ligand root mean square deviation (RMSD) analysis.

The average distance between the respective atom positions in the present configuration of a ligand and its reference configuration, which is typically the initial or crystal structure, is referred to as ligand RMSD. A lower RMSD value implies that the ligand’s structure is more stable and exhibits less movement, as it remains in close proximity to its reference state. It is a reliable method for evaluating the stability of a ligand and the conformational alterations that occur within a binding site. Maintaining a low and consistent RMSD during the simulation is indicative of a stable ligand, while variations in RMSD represent conformational shifts or instability. The mean Ligand RMSD scores for three selected compounds, comprising the CID: 359, 11640528, and 8768, were 0.069 **Å**, 2.027 **Å**, and 0.294 **Å**, respectively (Fig 8B). However, CID: 359 and 8768 exhibited significantly higher fluctuations compared to CID: 11640528, with continuous fluctuations observed throughout the 100 ns simulation period.

#### 3.7.3 Protein root mean square fluctuation (RMSF) analysis.

RMSF (Root Mean Square Fluctuation) analysis is utilized to quantify the dynamic interactions of the P-L complex, showing how each atomic localisation varies from its initial coordinates in a protein. Essentially, this measure highlights the ligand’s consistent stability with respect to the reference protein as calculated to visualise the location of the AA residue, which is often indicated using the spatial coordinates of the α carbon [72]. Consequently, the areas of lesser or higher flexibility across a protein framework for the chosen compounds are reflected in the mean RMSF scores, which range closer to 2–3 **Å** [73]. These include compounds CIDs: 359, 11640528, and 8768, which were 1.183 **Å**, 1.184 **Å**, and 1.288 **Å**, respectively (Fig 8C). The smallest and greatest RMSF scores of target protein were 0.465 **Å** and 9.079 **Å,** which were observed at the residual positions ASP 334 and MET 1, respectively. The RMSF values of the three ligands that were selected, CIDs: 359, 11640528, and 8768, showed the highest RMSF values at 8.089 **Å**, 10.883 **Å**, and 9.53 **Å**, respectively, all at the residual positions MET 1. The smallest values stood at 0.442 **Å**, 0.39 **Å**, and 0.485 **Å**, respectively, at the residual positions ASP 334, ASP 334, and SER 220. The selected compounds exhibited a peak area of fluctuations of the protein at ASP 290, ASN 67, GLY 167, GLY 104, GLY 152, GLY 158, and HIS 299, respectively. Moreover, the stiffest region of fluctuations was due to the secondary structural elements exhibiting the least flexibility, which would range from AA residues, followed by the region of AA residues. Maximum variation is seen at the beginning and end of the protein due to the presence of the β-sheet, α-helix, C-terminal, and N-terminal domains. Hence, for the CID: 8768 that is being studied, there is very little chance that an atom will change in real life.

#### 3.7.4 Radius of gyration (Rg) analysis.

The Rg (Radius of Gyration) of the P-L complex has been analysed to determine the protein’s stiffness and mobility. When predicting the structural activity of macromolecules, the computation of Rg is crucial since it is a key indicator of changes in complex compactness. Therefore, utilizing the Rg value over the 100 ns simulation length depicted in (Fig 8D), this study generated 1000 frames and employed a 100 ps interval for trajectory recording to analyse the Rg of three selected compounds, including CID: 359, CID: 11640528 and CID: 8768 in interaction with the target protein. The range of variations for CID: 359, CID: 11640528, and CID: 8768 was more consistent, as shown in Fig 8D. The minimum and maximum values were 2.064 **Å** in the 476 number frame to 2.148 **Å** in the 586 number frame (difference is 0.084 **Å**), 4.857 **Å** in the 292 frame to 7.782 **Å** in the 523 frame (difference is 3.212 **Å**), and 2.252 **Å** in the 639 frame to 2.382 **Å** in the 752 frame (difference is 0.13 **Å**), correspondingly. The average Rg scores for the compounds CID: 359, CID: 11640528, and CID: 8768 were 2.11 **Å**, 6.62 **Å**, and 2.32 **Å**, correspondingly.

#### 3.7.5 Solvent-accessible surface area (SASA) analysis.

SASA (Solvent Accessible Surface Area) is the surface region where solvent molecules engage with proteins or ligands, and during the simulation study, it reveals a correlation with the relationships that take place between the complex and the solvent [74]. Additionally, the folding of the protein was measured by computing the SASA, and proteins with greater folding rates have greater SASA scores [72]. Consequently, the mean SASA values of the protein in relation to the compounds CID: 359, 11640528, and 8768 were determined, which were 170.84 **Å**^2^, 283.22 **Å**^2^, and 91.85 **Å**^2^, as displayed in Fig 8E. The mean SASA score for the complex systems was found to be 90–285 **Å**^2^, which indicates that an amino acid residue within the complex procedure was frequently permeable to the targeted protein. According to the findings, the lowest SASA values of CID: 359, 11640528, and 8768 were 6.621 **Å**^2^, 134.33 **Å**^2^, and 4.678 **Å**^2^ in frame numbers 210, 2, and 968, respectively, and the highest were 283.964 **Å**^2^, 417.765 **Å**^2^, and 302.789 **Å**^2^ in frame numbers 704, 339, and 534, respectively.

#### 3.7.6 H-bonds analysis.

The maintenance of the protein structure and the binding of the protein to the ligand are both reliant on the H-bonds, which are principal relationships [74]. H-bonds serve a crucial role in stabilising the ligand with the target protein and contributing to regulating drug selectivity, metabolism, and adsorption [55]. A significant shift in the number of interprotein H-bonds is generally associated with an alteration in ternary protein configuration, and a large number of H-bonds between the protein and ligand indicate a higher degree of binding [71,74]. Consequently, during the 100 ns simulation period, the no. of H-bonds generated throughout the relationship between the P-L complex was observed, illustrated in Fig 8F. The mean H-bond scores for the compounds CID: 359, 11640528, and 8768 were reported to be 318.21, 313.68, and 309.35, respectively. A significant number of hydrogen bonds between 274 and 342 were formed simultaneously by each of the compounds over the simulation time. Hence, each compound will immensely enhance and sustain the P-L relationship.

#### 3.7.7 Protein-ligand (P-L) contact analysis.

**Fig 9:**
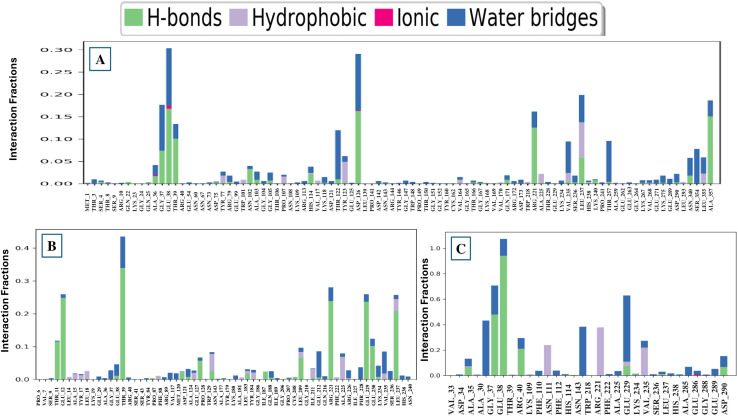
The P-L interactions observed throughout the 100 ns simulation are (A) CID: 359, (B) 11640528, and (C) 8768, shown here in complex with the target protein.

Protein-ligand interaction is an extensively used technique for investigating the molecular relationships between bound ligands and residues in a protein’s active sites [75]. Understanding the binding habits of compounds in the binding region of the receptor binding domain requires atomic-level investigation. The study of the binding pattern relies on intermolecular forces such as water bridges, ionic bonds, hydrophobic bonds, and hydrogen bonds [76]. These intermolecular forces of interaction were anticipated by employing simulation studies running for 100 ns. At the residues ALA 36, GLU 38, TYR 77, ARG 79, HIS 114, TYR 123, ASP 126, VAL 235, LEU 237, HIS 238, and LEU 355, CID: 359 generated multiple interactions with interaction fractions (IF) of 0.04, 0.32, 0.03, 0.02, 0.04, 0.52, 0.28, 0.10, 0.22, 0.02, and 0.07, correspondingly (Fig 9A). The specific interaction is continued during the simulation because of the repeated contacts between the identical subtype and the ligand. Multiple interactions with CID: 11640528 were generated by residues GLU 38 (0.05), MET 120 (0.03), TRP (0.08), LEU 183 (0.04), LEU 209 (0.11), ILE 211 (0.04), ARG 226 (0.03), LYS 234 (0.06), VAL 235 (0.10), and VAL 237 (0.28) (Fig 9B), while several interactions were made by the CID: 8768 at residues ALA (0.13), LYS 109 (0.31), VAL 235 (0.65), ALA 285 (0.07), and ASP 290 (0.09) (Fig 9C). However, CID: 359 and 11640528 exhibited a significantly higher affinity for interacting with the target protein in comparison to CID: 8768.

## 4. Discussion

The present study explores a novel approach to mitigating the impact of *Fusarium solani* infections in shrimp aquaculture by targeting the glutamine synthetase protein using metabolites derived from seaweed. Molecular docking methods determine the binding energetics of the predicted ligand-receptor complexes employing ranking values [77]. As previously mentioned, molecular docking was employed to screen 1,191 seaweed metabolites against the Glutamine synthetase (AF-Q9UUN6-F1-v4) of *F. solani*. It revealed strong binding affinities of the five selected (CID: 359, 11640528, 11568133, 8768, and 11672129) compounds to glutamine synthetase, with docking values of −5.752, −5.374, −5.166, −5.102, and −5.037 kcal/mol, correspondingly, and demonstrates the promising binding potential.

PK is an analysis of drug concentration variations over time in various body fluids, considering the properties of the medication in terms of ADME. Lipinski’s rule states the following conditions for an orally active drug: MW < 500 g/mol, consensus logP < 5, H-bond-donating atoms < 5, and H-bond-accepting atoms < 10 [78]. On the other hand, utilising technological developments like ProTox-3.0, which provides easy-to-obtain information from toxicity screening tools, offers an approach to carrying out *in-silico* toxicity investigation. According to the toxicity profiles of the three lead compounds (CID: 359, 11640528, and 8768), the *in silico* indicated low anticipated hazards. The inability of computational forecasts to adequately account for complicated toxicological behaviors, such as dose-dependent effects, chronic exposure, and possible off-target interactions, must be emphasized. Consequently, even though the compounds seem safe *in silico*, if *in vitro* or *in vivo* research reveals cytotoxicity or environmental persistence, their usefulness in aquaculture may be restricted. Before any possible therapeutic use, additional experimental analysis, such as cytotoxicity tests and shrimp bioassays, will be necessary to confirm their safety. Consequently, three compounds (CID: 359, 11640528, and 8768) were selected from the five compounds that displayed druggable PK characteristics and impressive safety profiles. Then, post-docking MM-GBSA was conducted to determine the negative binding energy of the P-L complex [41]. The negative binding free energies of the three chosen compounds were −16.27, −48.99, and −27.48 kcal/mol, respectively. To place these results in context, reported MM-GBSA binding free energies for known antifungal drugs such as fluconazole and itraconazole typically range from −15 to −55 kcal/mol, depending on the target and protocol [79,80]. Within this range, CID: 11640528 (−48.99 kcal/mol) shows binding comparable to itraconazole, CID: 8768 (−27.48 kcal/mol) indicates moderate affinity, and CID: 359 (−16.27 kcal/mol) reflects weaker binding. This comparison supports prioritizing CID: 11640528 and CID: 8768 for further validation, while noting that absolute MM-GBSA values are method-dependent and best used for relative ranking. In this study, CID: 11640528 and 8768 exhibited greater reactivity as a result of their HOMO-LUMO gap values, and QSAR analysis showed their biological activity, notably antifungal efficacy, rendering them more suitable as potential drug candidates.

MD simulations were used to analyse P-L relationships, durability, and structural modifications at various time periods, facilitating the identification of the most notable compounds for future investigation [81]. The three selected compounds, together with the target protein, have been assessed based on RMSD, ligand RMSD, RMSF, Rg, SASA values, H-bonds, and P-L interaction. The optimal durability of the compounds is displayed by the RMSD scores of the complex systems. The mean RMSD scores for CID: 359, 11640528, and 8768 were 3.364 **Å**, 2.484 **Å**, and 2.466 **Å**, correspondingly. The study indicates that CID: 11640528 and 8768 demonstrated excellent stability when complexed with the target protein, compared to CID: 359. The mean Ligand RMSD scores for three selected compounds, comprising the CID: 359, 11640528, and 8768, were 0.069 **Å**, 2.027 **Å**, and 0.294 **Å**, respectively. However, CID: 11640528 and 8768 exhibited significantly higher fluctuations compared to CID: 359. The RMSF analysis is employed to quantify the fluctuating interactions of the P-L complex, which exposes the changes in atomic localisation from the initial coordinates in a protein [82]. The mean RMSF values for the selected compounds CIDs: 359, 11640528, and 8768 were 1.183 **Å**, 1.184 **Å**, and 1.288 **Å**, correspondingly. These results suggest that the association of CID: 8768 induces minute alterations in protein structure. The three lead compounds were then examined based on Rg, SASA scores, H-bonds, and P-L interaction. The firmness and mobility of the proteins have been determined by analysing the Rg of the P-L complex [75]. The average Rg values for the compounds CID: 359, 11640528, and 8768 were 2.11 **Å**, 6.62 **Å**, and 2.32 **Å**, respectively. The surface area where solvent molecules interact with proteins or ligands is known as SASA [74]. The mean SASA values of the protein in association with the compounds CID: 359, 11640528, and 8768 were calculated, which were 170.84 **Å**^2^, 283.22 **Å**^2^, and 91.85 **Å**^2^. The H-bonds, which are fundamental relationships, are crucial for both the binding of the protein to the ligand and the preservation of the protein structure [74]. The average H-bond scores for the compounds CID: 359, 11640528, and 8768 were computed to be 318.21, 313.68, and 309.35, respectively.

Several computational assessments have been used in this study to analyse three compounds: CID: 359, 11640528, and 8768. In the docking and MD simulation study, CID: 8768 (Protocatechualdehyde) exhibits notable results. This compound has the best chance of preventing *Fusarium solani* infections in shrimp farming because it has the ability to inhibit the glutamine synthetase protein. Protocatechualdehyde, a seaweed metabolite, is present in *Polysiphonia lanosa* [64]*.* Previous investigations have demonstrated the possible therapeutic use of Protocatechualdehyde in the mitigation and cure of antioxidant activity [61] and anti-cancer [66], where we discovered its antifungal effectiveness in our research.

In order to verify the curative effects of potential drugs against target proteins, it is crucial to perform both *in vivo* and *in vitro* examinations. Further research is necessary for the final confirmation of compound-protein relationships, as limited resources could hinder comprehensive testing. This *in-silico* modelling advances the *in vitro* study carried out on various diseases, thereby enhancing the understanding and improving the development of therapeutics.

## 5. Conclusion

This study characterises seaweed metabolites as possible *F. solani* antifungals for shrimp aquaculture. No particular therapies exist for this widespread *F. solani*-caused illness, and our investigation explored novel glutamine synthetase protein inhibitors to eliminate fungal infections.

A diverse array of *in-silico* techniques was employed, and CID: 8768 (Protocatechualdehyde) was identified as the most promising therapeutic candidate due to its higher stability, which is present in *Polysiphonia lanosa* seaweed. However, these findings are based solely on computational predictions, and the candidate has not undergone clinical evaluation. Therefore, *in vitro* and *in-vivo* studies are essential to ascertain its efficacy. As a result, to validate Protocatechualdehyde as a potential therapeutic candidate for future applications, more experimental study is required to determine its effectiveness. We specifically propose a stepwise validation pipeline, beginning with in vitro enzyme inhibition assays against *F. solani* glutamine synthetase, followed by fungal growth inhibition and minimum inhibitory concentration (MIC) assays, and finally in vivo shrimp challenge experiments to confirm protective efficacy and safety under aquaculture conditions.

## 6. Future directions

While this study provides a comprehensive in silico analysis of seaweed-derived metabolites as potential inhibitors of *Fusarium solani* glutamine synthetase, experimental validation is essential to translate these findings into practical applications. As a next step, we propose a structured pipeline of investigations. First, *in vitro* enzyme inhibition assays should be conducted to confirm direct binding and inhibitory effects on *F. solani* glutamine synthetase. Second, antifungal growth assays, including determination of minimum inhibitory concentrations (MIC), should be performed to assess the biological efficacy of the identified compounds. Finally, *in vivo* shrimp challenge experiments are recommended to evaluate protective efficacy, immune responses, and safety under aquaculture conditions. Implementing these studies will provide critical evidence to validate the therapeutic potential of the lead compounds identified through computational methods.

## Supporting information

S1 File Docking results. (CSV)

S2 File Post docking MM-GBSA. (CSV)

S3 File RMSD analysis. (XLSX)

S4 File RMSF analysis. (XLSX)

S5 File Ligand properties. (XLSX)
